# A new pragmatic design for dose escalation in phase 1 clinical trials using an adaptive continual reassessment method

**DOI:** 10.1186/s12885-019-5801-3

**Published:** 2019-06-26

**Authors:** Bernard North, Hemant Mahendrakumar Kocher, Peter Sasieni

**Affiliations:** 1 Cancer Prevention Trials Unit, Wolfson Institute of Preventive Medicine, Queen Mary University of London, London, UK; 2 Centre for Tumour Biology and Experimental Cancer Medicine, Barts Cancer Institute- a CRUK Centre of Excellence, Queen Mary University London, London, EC1M 6BQ UK; 3 Current addresses: Exploristics Ltd, Belfast, UK; 4 Current addresses: School of Cancer & Pharmaceutical Sciences, and King’s Clinical Trials Unit, King’s College London, London, UK

**Keywords:** Adaptive design, Bayesian adaptive, Simulation, 3 + 3 design, Maximal tolerated dose, Dose-finding, CRM, Model-based design, Rule-based design

## Abstract

**Background:**

A key challenge in phase I trials is maintaining rapid escalation in order to avoid exposing too many patients to sub-therapeutic doses, while preserving safety by limiting the frequency of toxic events. Traditional rule-based designs require temporarily stopping recruitment whilst waiting to see whether enrolled patients develop toxicity. This can be both inefficient and introduces logistic challenges to recruitment in the clinic. We describe a novel two-stage dose assignment procedure designed for a phase I clinical trial (STARPAC), where a good estimation of prior was possible.

**Methods:**

The STARPAC design uses rule-based design until the first patient has a dose limiting toxicity (DLT) and then switches to a modified CRM, with rules to handle patient recruitment during follow-up of earlier patients. STARPAC design is compared via simulations with the TITE-CRM and 3 + 3 methods in various toxicity estimate (T1–5), rate of recruitment (R1–2), and DLT events timing (DT1–4), scenarios using several metrics: accuracy of maximum tolerated dose (MTD), numbers of DLTs, number of patients enrolled and those missed; duration of trial; and proportion of patients treated at the therapeutic dose or MTD.

**Results:**

The simulations suggest that STARPAC design performed well in MTD estimation and in treating patients at the highest possible therapeutic levels. STARPAC and TITE-CRM were comparable in the number of patients required and DLTs incurred. The 3 + 3 design often had fewer patients and DLTs although this is due to its low escalation rate leading to poor MTD estimation. For the numbers of declined patients and MTD estimation 3 + 3 is uniformly worse, with STARPAC being better in those metrics for high toxicity scenarios and TITE-CRM better with low toxicity. In situations including doses with toxicities both above and below 30%, the STARPAC design outperformed TITE-CRM with respect to every metric.

**Conclusion:**

When considering doses with toxicities both above and below the target of 30% toxicities, the two-stage STARPAC dose escalation design provides a more efficient phase I trial design than either the traditional 3 + 3 or the TITE-CRM design. Trialists should model various designs via simulation to adopt the most efficient design for their clinical scenario.

**Trial registration:**

Clinical Trials NCT03307148 (11 October 2017).

**Electronic supplementary material:**

The online version of this article (10.1186/s12885-019-5801-3) contains supplementary material, which is available to authorized users.

## Background

Phase I clinical trials are an essential early-stage investigation in the development of anti-cancer and other therapeutic drugs. The main goal of these studies is to identify the appropriate dose for new drugs or drug combinations for phase II trials, often called the recommended phase 2 dose (RP2D). These studies typically involve a small number of patients. A key principle for dose escalation in phase I trials is maintaining rapid dose-escalation in order to avoid exposing too many patients to sub-therapeutic doses while preserving safety by limiting the frequency of toxic events (dose limiting toxicities or DLTs). The maximum tolerated dose (MTD) is estimated limiting the probability of a DLT to a particular level, the target toxicity level (TTL), which is often set at 30%. Dose escalation methods for phase I cancer clinical trials fall into two broad classes: the traditional and often used rule-based, or “up and down”, designs, which include the traditional 3 + 3 design [1, 2] and its variations; and relatively recent, model-based designs such as the continual reassessment method (CRM) [3].

The most commonly employed rule-based design is the 3 + 3 design. It sequentially enrols cohorts of three patients; the first cohort is treated at a starting dose that is considered to be safe based on extrapolation from animal toxicological data or prior experience in other disease conditions, and the subsequent cohorts are treated at increasing dose levels that have been fixed in advance. The 3 + 3 is conservative with respect to the number of toxicities which occur, because the dose escalation is performed with caution, but it can potentially lead to a large number of patients needed to estimate the MTD, especially if the true MTD is located in the upper range of the doses tested. This method has been criticized for assigning low, possibly sub-therapeutic, doses to a high proportion of patients, often only making use of information given by the last three or six patients enrolled; thereby providing an inefficient estimation of the MTD and inflexibility in that the method is tailored to a target toxicity level of around 30%.

An alternative to the rule-based methods for finding the MTD is to use a model-based approach that assumes there is a monotone relationship between the dose and the probability of a DLT. The most commonly applied model-based approach is the CRM and its variants. The CRM pre-specifies a dose-toxicity curve (DTC) as well as the TTL. Prior estimates of the probability of a DLT at each dose level are provided based on clinical experience. The DTC is updated with accumulating toxicity data from the trial. New patients are given the MTD derived from the updated DTC.

The CRM method in its original form treated patients individually with the initial patient dosed at the MTD suggested by the (possibly inaccurate) prior DTC, allowed dose escalations of more than one level (dose skipping), and required a fixed number of patients (usually around 20). This caused some criticism both on the grounds of excess toxicity exposure and the length of the study given toxicity data from previous patients may take time to occur. Modified designs treat the initial patient at a low level and do not allow dose-skipping. They may also treat patients in cohorts of more than one, and include early stopping rules to limit study duration [4]. Another suggestion to limit toxicity is the *escalation with overdose control method* [5].

A further class of models designed to address the issue of limited information in the early stage of a CRM are the two-stage designs [6, 7] whereby patients are initially treated according to some rule-based design with a transition to the CRM approach often on occurrence of the first DLT.

### Relative performance of the CRM and 3 + 3 method

Both the 3 + 3 and the CRM method with its variants have advantages and disadvantages depending on the toxicity profile of the drug, the number of dose levels, the DLT required, and the accuracy of the prior estimate of the CRM dose-toxicity curve [8, 9]. Although ruled-based designs, compared with model-based designs, tend to have lower probability of finding the true MTD and to have more patients treated at sub-MTD doses with potentially less therapeutic value, they are likely to have fewer toxicities and can, sometimes, require fewer patients. These four metrics: MTD accuracy, patients dosed at MTD, low toxicity and economy of patient numbers are important in comparing the performance of alternative phase 1 designs. Van Brummelen et al., 2016 reported that (*n* = 11) model-based trials were shorter, requiring fewer patients, incurred a lower percentage of DLTs and treated fewer patients at potentially sub-therapeutic levels compared with (*n* = 161) rule-based trials.

Recent (UK) Medical Research Council (MRC) [10], Committee for Medicinal Products for Human Use (CHMP), (CHMP 2006) and FDA (FDA 2011) guidance recommends that alternative to the 3 + 3 designs should be considered. Despite these design modifications and recommendations, the CRM and its variants have not been widely adopted with up to 94% of studies (*n* = 172) following a rule-based design. (van Brummelen et al., 2016).

Love (2017) found that the most prominent barriers to implementation of a model-based design (e.g. CRM) were lack of suitable training, chief investigators’ preference for rule-based designs (e.g., 3 + 3), a mistaken belief in regulatory preference for rule-based designs and limited resources for study design before funding especially when DLT may occur in a delayed fashion (up to few weeks after initial administration) or is cumulative after repeated administration of a drug.

### Accrual given incomplete DLT observation

A challenge of using both rule-based and model-based methods is that a toxicity/DLT may not be observed for some time after a patient is recruited. In cancer trials it is common to wish to consider DLT’s observed during the first cycle of treatment (usually 14–28 days). Even when using cohorts of three patients at the same dose, this can result in patients being recruited while previous patients have only partial follow-up and therefore, with incomplete toxicity responses. This is particularly a problem early in a trial when the next patient could be recruited and require a dose recommendation, when no previous patients have completed their first cycle to estimate full toxicity. There have been several attempts to address this both in the context of rule-based and model-based studies. An alternative rule-based design, [11], the accelerated titration design, treats one patient per dose level until a patient experiences a DLT at which point the traditional 3 + 3 method is employed. Another rule-based approach, the rolling six design [12], allows for accrual of two to six patients on the same dose which is determined by the number of patients currently enrolled and evaluable, the number experiencing dose-limiting toxicity (DLT), and the number still at risk of developing a DLT. Within the CRM context one of the modifications previously suggested [4] was to recruit patients in cohorts rather than singly to increase accrual. A modification to the CRM process was proposed [13] called the time-to-event continual reassessment method (TITE-CRM), that allows patients to be entered in a staggered fashion enrolling new patients while existing patients have an incomplete observation by incorporating the time to the event (the event being a DLT) or the partial follow-up without DLT for each patient. This method can be an efficient method of allowing the advantages of a CRM design to be used with partial follow-up. However, early in the study, unless overdose control is employed or dose-skipping prevented, this method can lead to increased DLTs [14].

In this article we propose an alternative to TITE-CRM (in order to overcome its limitations) for the STARPAC trial by using a hybrid two-stage dose escalation with an initial stage of accelerated dose escalation until the occurrence of a DLT at which point an amended CRM is employed. We then compare the STARPAC design proposal with standard 3 + 3 design, and TITE-CRM using ~ 1000 simulations per scenario to evaluate the key metrics of these designs: patients required and skipped (patient burden), DLTs encountered (toxicity), study length (economy), MTD determination (accuracy), patients dosed at MTD (therapeutic utility).

## Methods

### The STARPAC trial

STARPAC is a Phase 1 trial of repurposing all trans-retinoic acid (ATRA) as a stromal targeting agent for pancreatic cancer alongside gemcitabine and nab-Paclitaxel. STARPAC patients have histologically proven pancreatic ductal adenocarcinoma (PDAC) which is locally advanced or metastatic disease which is measurable according to the Response Evaluation Criteria in Solid Tumours (RECIST v1.1). Pancreatic cancer is the fourth-highest cancer killer worldwide (~ 310,000 patients), responsible for 6% of cancer deaths (overall median survival ~ 3 months). One of the characteristics of pancreatic cancer is its intense desmoplastic stroma, which can account for up to 70% of the tumour volume and actively participates in tumour initiation, progression, metastases and the response to therapy [15]. The formation of stroma is driven by pancreatic stellate cells (PSCs) as they change from a quiescent, vitamin A storing phenotype to an activated myofibroblast-like cell. Normalising the tumour stroma by reprogramming PSCs to their quiescent phenotype by all-trans retinoic acid (ATRA) [16, 17], and thereby restoring a more physiological secretome, is an attractive approach to be explored with this trial. The combination of gemcitabine-nab-Paclitaxel is licenced for treatment of pancreatic cancer with well-known toxicity profile [18]. ATRA is also a well-established drug with more than four decades of clinical experience for other cancers [19–22], and its analogues, such as 13-cis retinoic acid (13cisRA)) have been used in context of pancreatic cancer [23–25]. However, the combination of these three drugs has never been used in pancreatic cancer, and hence the phase I clinical trial. The toxicities are well-known and are hypothesised for the purposed of design to be additive and non-synergistic based on the knowledge of mode of action. Therefore, five dose levels were considered, to ensure that no patients are treated at sup-optimal cytotoxic chemotherapy level. Lowest combination of cytotoxic chemotherapy was chosen at 80% because that was median dosing intensity in phase III clinical trial [26]. Hence we have designed the STARPAC trial with five dose levels, D1-D5, of Gemcitabine, Nab-Paclitaxel and ATRA identifies the MTD (maximum tolerated dose, Table 1).

**Table 1:** Dose Levels of STARPAC clinical trial

Dose level	Gemcitabine		Nab-Paclitaxel		ATRA	
D1	80%	800 mg/m^2^	80%	100 mg/m^2^	33%	15 mg/m^2^
D2	80%	800 mg/m^2^	80%	100 mg/m^2^	66%	30 mg/m^2^
D3	80%	800 mg/m^2^	80%	100 mg/m^2^	100%	45 mg/m^2^
D4	100%	1000 mg/m^2^	80%	100 mg/m^2^	100%	45 mg/m^2^
D5	100%	1000 mg/m^2^	100%	125 mg/m^2^	100%	45 mg/m^2^

### The STARPAC design and rules

We implemented a two-stage dose selection in order to balance prevention of excessive toxicity with the objective of maintaining rapid dose escalation when justified as described before [27], where rule-based design (Stage 1) was used with a switch to a model-based dose escalation algorithm (Stage 2) upon occurrence of the first DLT. The first three patients are assigned to dose level D2 unless one of those three patients incurs a DLT when the switch to the CRM-based Stage 2 will occur. The dose selection design is implemented using the R programming language with the CRM component using the *bcrm* package [28] (Additional file 1).

Since DLTs usually manifest after two weeks [26], STARPAC Stage 1 uses two key rules to determine dose escalation for patients, when no DLT occurs:

- If a patient has received two-weeks of treatment on the current dose level, the next patient to be recruited will be allocated to the next higher dose level.
- If no patient has received two-weeks of treatment on the current dose level, the next patient to be recruited will be dosed on the same dose level.

On occurrence of the first DLT, Stage 2 uses a CRM model using data from patients who have had a DLT or more than three weeks on study treatment without a DLT. The CRM uses Bayesian methodology based on a power function dose toxicity curve, using physician experience to estimate the probability of toxicity occurring at each dose level D1-D5 as 10, 15, 20, 25 and 30% respectively [24–26]. We use the hyperbolic tangent parameter has the suggested initial lognormal prior distribution with mean zero and variance 1.34^2^ [29]. As patient toxicity data is accumulated alongside their assigned doses, this is input to the modified CRM, which will then recommend a dose level for the next patient employing a dose-skipping restriction [30].

The study stopping rule was defined when 6 patients are recruited to the same dose throughout either stage or when 24 patients are recruited in total. At that point a CRM analysis will be performed on the data when all patients have completed their first cycle (28 days). The final MTD will be defined as the highest dose level of ATRA and Gemcitabine / Nab-Paclitaxel for which the CRM estimated probability of a patient experiencing a DLT is closest to 30%. The design also allows us to switch from Stage 2 back to Stage 1 in the event that a possible DLT is later determined not to have been a DLT.

As a further safety measure, we also ensure that a maximum of three patients are recruited (on the same dose) in any two-week period. For example, if first patient three are recruited by week 4 (on say dose level D2), and next two patients are recruited in week 5 (on D3), then if patient 6 arrives in week 5 they can be recruited (on D3) provided there have been no DLTs. We will allow a third patient in a 2-week period to wait up to seven days before start of treatment in order for this condition not to be violated. If more than seven days is required before a patient becomes eligible then the patient is not recruited to the trial and is allocated to standard care.

**Fig. 1:**
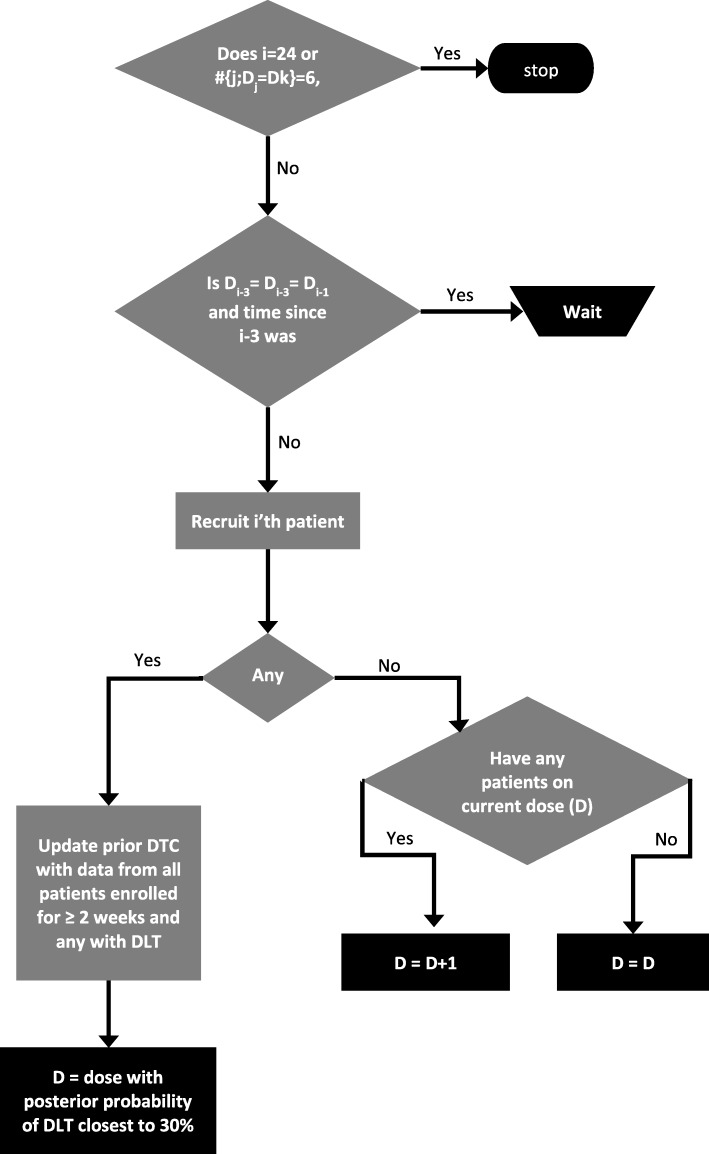
Flowchart of STARPAC design

The STARPAC design assigns first patient to dose level D2. If no DLT has been observed, next patient is assigned to either the same dose as the previous patient, if no patient on that dose has received at least 2 weeks of treatment; or, the next dose if at least one patient has already received 2 weeks of treatment at that dose. However, once a DLT has been observed, the next dose is assigned using a model-based CRM taking into account all DLTs and all patients who have had at least 3 weeks of treatment without a DLT. We use a power function dose toxicity curve and do not allow dose-skipping – if the dose is to be increased it will only do so by one level. We will stop recruitment when 6 patients have recruited to the same dose or when the 24th patient is recruited. Once all patients have completed 28 days of follow-up, the MTD is selected as that with a posterior probability of a DLT that is closest to the TTL. This is illustrated in Fig. 1.

### Simulations

The properties of the STARPAC design were assessed with simulation by the R programming language [31] using the *bcrm* function (Additional file 1). Hypothetical data was simulated with plausible various scenarios, based on clinical experience [18] (Table 2). The STARPAC design was compared to both the TITE-CRM procedure and the classic 3 + 3 procedure.

**Table 2:** Scenarios for simulation

Toxicity scenario	Type of scenario	Dose Levels				
		D1	D2	D3	D4	D5
T1	Linear, very high	10	20	**30**	40	50
T2	Linear, high	10	20	25	**30**	40
T3	Linear, anticipated	10	15	20	25	**30**
T4	Linear, low	5	10	12	15	20
T5	Non-linear, variable	5	15	**30**	50	70

1. Rates of recruitment scenarios (R): patients recruited randomly, with two (Poisson process) arrival rates considered:

a. R1: routine average arrival 1 patient per week and

b. R2: accelerated average arrival rate 1.5 patients per week.

2 Toxicity occurrence scenarios (T)

3. The DLT times (for those with a DLT) were simulated using 4 timing (DT) scenarios:

a. DT1: uniformly between 8 and 21 days after recruitment: most plausible based on clinical experience [16]

b. DT2: uniformly between 11 and 28 days after recruitment

c. DT3: at either 11 or 21 days with probabilities of 25 and 75%

d. DT4: at either 11 or 21 days with probabilities 30 and 70%.

Bold typeface signifies acceptable DLT to declare MTD.

### Suppositions applied to TITE-CRM

TITE-CRM was applied to the simulations using the logistic form for the DTC and starting with the first 3 patients at D2 unless a DLT occurs. The *titecrm* function of the *dfcrm* R package [32] was used for assigning doses to the simulated patients. For the TITE-CRM procedure the safety measure of a maximum of three patients recruited in any two-week period was also applied.

### Suppositions for 3 + 3 design

The 3 + 3 method was also applied starting at D2, with excessive toxicity at a dose leading to dose de-escalation. If the lower dose has been evaluated before, it is declared to be the (estimated) MTD. However, if the lower dose has not been evaluated then three patients are recruited. If all doses are declared too toxic by 3 + 3, then no MTD is declared. For the 3 + 3 design, presenting patients were not recruited if the three patients currently being treated had not yet provided a toxicity result enabling the dose level of the next three patients to be determined. A toxicity result is available when the current 3 patients complete their cycle without any DLTs (at which point the next three can be recruited at a higher dose level) or with 1 DLT (at which point the next three can be recruited at the same dose level), or when a second DLT occurs (at which point the next three can be recruited at a lower dose level) or if for a second set of three patients (after a previous single DLT) when a single DLT occurs (at which point the next three can be recruited at a lower dose level). Any patients presenting while the next dose level is undetermined are unable to be recruited.

1000 datasets were simulated using each of the three designs and for each of the forty (toxicity (T) and DLT timing (DT)) scenarios using the different patient arrival rates (R).

These in silico simulations did not require ethical approval, as no humans or animals were used. STARPAC study was approved by South Central - Berkshire Research Ethics Committee (15/SC/0548), but is not part of the manuscript.

## Results

### Simulation results

Summary of performance based on simulations for STARPAC, TITE-CRM and 3 + 3 designs is presented in terms of: patients required and skipped (not-recruited due to insufficient information to determine next dosing level: patient burden), DLTs encountered (toxicity), study length (economy), MTD determination (accuracy), patients dosed at MTD (therapeutic utility) in Table 3.

**Table 3:** Results of simulations

Design	toxicity	patients	DLTs	study length (1.0/wk)	number skipped (1.0/wk)	study length (1.5/wk)	number skipped (1.5/wk)	MTD
STARPAC	T1	12.2 (12.2, 12.7)	3.28 (3.20, 3.54)	15.8 (15.8, 16.1)	3.47 (3.47, 3.76)	13.2 (13.0, 13.6)	7.32 (7.32, 7.67)	0.342 (0.317, 0.363)
TITE-CRM	T1	14.5 (14.5, 15.3)	4.23 (4.23, 4.66)	19.1 (19.1, 19.9)	4.27 (4.27, 4.57)	16.2 (15.9, 16.3)	9.07 (9.07, 9.41)	0.217 (0.162, 0.217)
3 + 3	T1	10.2 (10.1, 10.4)	2.64 (2.62, 2.71)	20.9 (20.9, 22.2)	9.90 (9.90, 10.28)	17.9 (17.9, 19.0)	14.44 (14.41, 14.95)	0.234 (0.234, 0.260)
STARPAC	T2	12.5 (12.4, 13.2)	3.01 (2.97, 3.30)	16.2 (16.2, 16.5)	3.70 (3.67, 3.75)	13.2 (13.2, 14.1)	7.57 (7.50, 8.09)	0.163 (0.117, 0.174)
TITE-CRM	T2	14.0 (13.8, 14.3)	3.66 (3.59, 3.81)	18.3 (17.9, 18.3)	4.15 (4.04, 4.15)	15.4 (14.9, 15.4)	8.90 (8.43, 8.95)	0.118 (0.070, 0.118)
3 + 3	T2	11.1 (11.1, 11.1)	2.59 (2.59, 2.63)	23.2 (23.2, 24.2)	10.95 (10.95, 11.17)	19.7 (19.7, 20.7)	16.05 (16.05, 16.31)	0.127 (0.127, 0.144)
STARPAC	T3	13.2 (13.0, 13.7)	2.74 (2.68, 2.90)	17.1 (17.1, 17.4)	3.86 (3.82, 4.11)	13.9 (13.9, 14.8)	7.87 (7.87, 8.38)	0.468 (0.441, 0.506)
TITE-CRM	T3	13.0 (12.7, 13.2)	2.88 (2.85, 3.02)	17.0 (16.3, 17.0)	3.87 (3.71, 3.87)	14.0 (13.7, 14.1)	8.00 (7.90, 8.12)	0.637 (0.637, 0.708)
3 + 3	T3	11.9 (11.9, 12.2)	2.39 (2.39, 2.43)	25.3 (25.3, 27.1)	12.18 (12.18, 12.67)	21.6 (21.6, 23.2)	17.93 (17.93, 18.75)	0.165 (0.141, 0.168)
STARPAC	T4	15.0 (14.7, 15.6)	2.23 (2.16, 2.37)	19.5 (19.4, 19.7)	4.60 (4.58, 4.64)	15.8 (15.8, 16.8)	8.90 (8.90, 9.69)	0.728 (0.691, 0.776)
TITE-CRM	T4	11.4 (10.9, 11.6)	1.71 (1.63, 1.82)	14.6 (14.6, 15.0)	3.32 (3.19, 3.40)	12.3 (11.8, 12.5)	7.04 (6.59, 7.04)	0.895 (0.879, 0.932)
3 + 3	T4	13.2 (13.2, 13.4)	1.79 (1.78, 1.82)	29.2 (29.2, 29.9)	14.12 (14.12, 14.84)	24.9 (24.9, 25.7)	20.81 (20.81, 21.85)	0.456 (0.443, 0.473)
STARPAC	T5	12.8 (12.7, 13.3)	3.59 (3.54, 3.81)	16.5 (16.4, 17.0)	3.74 (3.74, 3.88)	13.7 (13.6, 14.2)	7.41 (7.41, 8.08)	0.444 (0.439, 0.476)
TITE-CRM	T5	16.6 (16.5, 17.0)	5.11 (5.11, 5.51)	21.6 (21.5, 22.3)	5.08 (4.92, 5.17)	18.2 (17.9, 18.6)	10.66 (10.44, 10.72)	0.347 (0.255, 0.347)
3 + 3	T5	9.9 (9.9, 10.1)	2.65 (2.65, 2.68)	20.4 (20.4, 21.7)	9.55 (9.55, 9.98)	17.3 (17.3, 18.7)	13.91 (13.91, 14.68)	0.349 (0.332, 0.349)

We also report the proportion of times each dose level is selected as MTD and the proportion of patients treated at each dose (Additional file 2).

In Table 3 data are summarised for the three designs in each of the five toxicity (T) scenarios. Values in Table 3 are for DLT timing-scenario DT1. Results for the study length and the numbers of patients skipped are presented separately depending on the recruitment rate. For all other results, the value in Table 3 is for R1 (one patient per week). Below each value in Table 3 is the range of that parameter across the four DLT timing scenarios (DT1-DT4) and (except for study length and patients skipped) the two recruitment rates. It is interesting to note that the ranges are narrow compared to the differences between designs and toxicity scenarios, suggesting that DLT timing scenarios has limited impact on any of the designs, an important consideration for future designs.

### Accuracy to determine MTD

The classic, rule-based 3 + 3 design never accurately estimates the correct MTD. For the scenarios including doses with greater than 30% toxicity (T1 and T2), STARPAC design is more likely to select the correct MTD than the other two designs, but all fair poorly. For those with the maximal dose included to be less than or equal to 30% (T3 and T4), TITE-CRM is more likely to pick the correct MTD, than STARPAC design. In these scenarios (T3 and T4), STARPAC is much more likely to pick the correct MTD than 3 + 3. We note that the likelihood of identifying the MTD with any of these designs is highly variable depending on the underlying toxicities of the doses considered. For example, in scenario T2, the MTD is only correctly identified less than 20% of the time (even by the best design). For scenario T4 in which the maximal dose has toxicity of just 20%, TITE-CRM correctly chooses that does up to 90% of the time, and STARPAC over 70%. In the highly variable, non-linear scenario, STARPAC design outperforms the other two designs.

### Toxicity (DLT frequency)

None of the designs has more than average six patients with DLTs in any of the scenarios studied, and mostly the mean is 3–4 DLTs per trial. For four of the five toxicity rate scenarios, 3 + 3 has the fewest patients and the fewest DLTs, but, as noted, it is also least likely to select the target dose. We must note that a design that stops after treating three patients at the lowest dose will always recruit the fewest and have the fewest DLTs, but it does not help one find the MTD. TITE-CRM design has the most DLTs in all scenario compared to other design, except for T4 scenario where STARPAC design fairs poorly.

### Patients required and study length (economy)

The ordering of designs in terms of the number of patients recruited is always the same as for the frequency of DLTs within the trial. On the other hand, the rule-based 3 + 3 design most often is longest in study length, but requires least patients except for scenario T4 where it requires more patients than TITE-CRM (but fewer than STARPAC design). TITE-CRM requires more patients than STARPAC design for scenarios T1, T2 and T5, whereas STARPAC requires more for T4 (and there is little to choose between the designs for T3).

### Skipped patients (patient and investigator burden)

For the numbers of skipped patients 3 + 3 is uniformly (and substantially) worse than the other designs. STARPAC is best in scenarios T1, T2 and T5; whereas TITE-CRM is best for T4; and the two designs are very similar for T3. The order of designs in terms of the study duration is always the same as for patients skipped – the more patients are skipped the longer the trial.

## Discussion

**Fig. 2:**
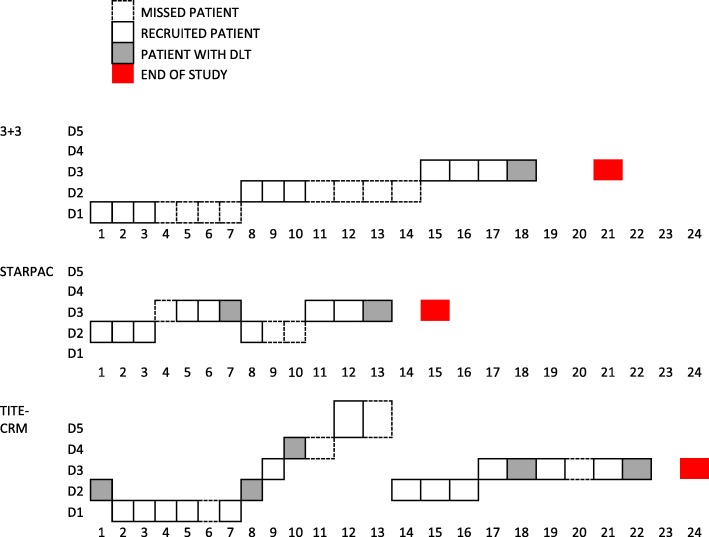
Schematic of recruitment of patients in STARPAC, 3 + 3 and TITE-CRM designs of a hypothetical scenario where the true toxicity is T1, recruitment rate is R1 and DLT timings are DT4. Missed patients are with dashed boxes, recruited patients are with solid boxes and patients with DLT (which may happen anytime between days 7 to 21 as for DT4) are shown in grey boxes

It has been noted that phase 1 trials often have a correct MTD estimation rate of only around 40% due to the low sample size [33]. We have described an alternative two stage dose-escalation (STARPAC) adaptive design [30] for phase 1 trials with delayed toxicity estimation (up to 28 days). We compare STARPAC design with TITE-CRM and 3 + 3 designs via simulation with the R programming language to illustrate the utility of simulation-based assessment under various plausible clinical scenarios, to enable evidenced based judgement dependent on key metrics which may relevant for the disease and patient population. An illustration of comparison is shown in Fig. 2. Our design compares favourably to other recently described modifications of TITE-CRM [34–37], such that recruitment is quicker and application of the design is easier. Furthermore, we have applied this design after rigorous simulations on to an actual oncology clinical trial, whilst others have yet to find real-life application.

Our simulations suggest that accuracy depends critically on the real toxicities of the doses studied, an important clinical consideration. Nevertheless, we have seen that in our five scenarios it varied between just over 10% to just under 90%, across the three design models and multiple scenarios that we have compared.

## Conclusions

The STARPAC design has advantages and disadvantages compared to the alternative TITE-CRM method, and both methods appear superior to the traditional 3 + 3 design particularly with regard to correct estimation of the MTD and duration of the trial. However, whenever the doses considered for the trial include ones with toxicity levels both above and below the target (TTL) of 30%, the new STARPAC design outperforms both 3 + 3 and TITE-CRM.

There are several reasons suggested for the high failure rate of confirmatory phase 3 trials. One reason may be a suboptimal treatment dose being selected for phase 2 and phase 3 trials. In our simulations the proposed STARPAC design is nearly always superior to 3 + 3 in terms of the accuracy of MTD estimation and, in studies that include doses with toxicity levels above 30%, it is superior to TITE-CRM as well.

We encourage clinical trialists to use simulations to provide evidence based adoption of different design models with particular reference to disease condition and patient population being studied.

## Additional files

Additional file 1: STARPAC programming package: Code in R using a modified brcm package for STARPAC design. - Description of data: word document with Program code. (TXT 11 kb)

Additional file 2: Detailed STARPAC Tables: Detailed results of all simulations at all toxicity scenarios (T1-T5), rates of recruitment (R1-R2) and DLT timing scenarios (DT1–4) - Description of data: excel spreadsheet with multiple tabs. (DOCX 27 kb)

## Data Availability

Raw R program file is available as Additional file 1 for use by other researchers. Summary raw data is available in Additional file 2.

## Abbreviations

13-cisRA: 13-cis retinoic acid
ATRA: All trans retinoic acid
CRM: Continual reassessment method
DLT: Dose limiting toxicities
DT: DLT timing
DTC: Dose-toxicity curve
MTD: Maximum tolerated dose
R: Rates of recruitment
RP2D: Recommended phase 2 dose
T: Toxicity
TITE-CRM: Time-to-event continual reassessment method
TTL: Target toxicity level
