# Supplementary material for: A new pragmatic design for dose escalation in phase 1 clinical trials using an adaptive continual reassessment method
Source: BMC Cancer. 2019 Jun 26;19:632. doi: 10.1186/s12885-019-5801-3 (PMC6595589; doi:10.1186/s12885-019-5801-3)
Supplement: Supplementary file 2 — Detailed STARPAC Tables: Detailed results of all simulations at all toxicity scenarios (T1-T5), rates of recruitment (R1-R2) and DLT timing scenarios (DT1–4) - Description of data: excel spreadsheet with multiple tabs. (DOCX 27 kb) [file 12885_2019_5801_MOESM2_ESM.docx]

**Table 1: Dose Levels of STARPAC clinical trial**

| **Dose level** | **Gemcitabine** | | **Nab-Paclitaxel** | | **ATRA** | |
| --- | --- | --- | --- | --- | --- | --- |
| **D1** | 80% | 800 mg/m^2^ | 80% | 100 mg/m^2^ | 33% | 15 mg/m^2^ |
| **D2** | 80% | 800 mg/m^2^ | 80% | 100mg/m^2^ | 66% | 30 mg/m^2^ |
| **D3** | 80% | 800 mg/m^2^ | 80% | 100 mg/m^2^ | 100% | 45 mg/m^2^ |
| **D4** | 100% | 1000 mg/m^2^ | 80% | 100 mg/m^2^ | 100% | 45 mg/m^2^ |
| **D5** | 100% | 1000 mg/m^2^ | 100% | 125 mg/m^2^ | 100% | 45 mg/m^2^ |

**Table 2: Scenarios for simulation.**

1. Rates of recruitment scenarios (R): patients recruited randomly, with two (Poisson process) arrival rates considered:
   1. R1: routine average arrival 1 patient per week and
   2. R2: accelerated average arrival rate 1.5 patients per week.
2. Toxicity occurrence scenarios (T)

| Toxicity scenario | Type of scenario | Dose Levels | | | | |
| --- | --- | --- | --- | --- | --- | --- |
|  |  | D1 | D2 | D3 | D4 | D5 |
| T1 | Linear, very high | 10 | 20 | **30** | 40 | 50 |
| T2 | Linear, high | 10 | 20 | 25 | **30** | 40 |
| T3 | Linear, anticipated | 10 | 15 | 20 | 25 | **30** |
| T4 | Linear, low | 5 | 10 | 12 | 15 | 20 |
| T5 | Non-linear, variable | 5 | 15 | **30** | 50 | 70 |

1. The DLT times (for those with a DLT) were simulated using 4 timing (DT) scenarios:
2. DT1: uniformly between 8 and 21 days after recruitment: most plausible based on clinical experience [16]
3. DT2: uniformly between 11 and 28 days after recruitment
4. DT3: at either 11 or 21 days with probabilities of 25% and 75%
5. DT4: at either 11 or 21 days with probabilities 30% and 70%.

**Table 3: Results of simulations**

| **Design** | **toxicity** | **patients** | | **DLTs** | | **study length (1.0/wk)** | | **number skipped (1.0/wk)** | | **study length (1.5/wk)** | | **number skipped (1.5/wk)** | | **MTD** | |
| --- | --- | --- | --- | --- | --- | --- | --- | --- | --- | --- | --- | --- | --- | --- | --- |
| **STARPAC** | **T1** | 12.2 | | 3.28 | | 15.8 | | 3.47 | | 13.2 | | 7.32 | | 0.342 | |
|  |  | (12.2, | 12.7) | (3.20, | 3.54) | (15.8, | 16.1) | (3.47, | 3.76) | (13.0, | 13.6) | (7.32, | 7.67) | (0.317, | 0.363) |
| **TITE-CRM** | **T1** | 14.5 | | 4.23 | | 19.1 | | 4.27 | | 16.2 | | 9.07 | | 0.217 | |
|  |  | (14.5, | 15.3) | (4.23, | 4.66) | (19.1, | 19.9) | (4.27, | 4.57) | (15.9, | 16.3) | (9.07, | 9.41) | (0.162, | 0.217) |
| **3+3** | **T1** | 10.2 | | 2.64 | | 20.9 | | 9.90 | | 17.9 | | 14.44 | | 0.234 | |
|  |  | (10.1, | 10.4) | (2.62, | 2.71) | (20.9, | 22.2) | (9.90, | 10.28) | (17.9, | 19.0) | (14.41, | 14.95) | (0.234, | 0.260) |
| **STARPAC** | **T2** | 12.5 | | 3.01 | | 16.2 | | 3.70 | | 13.2 | | 7.57 | | 0.163 | |
|  |  | (12.4, | 13.2) | (2.97, | 3.30) | (16.2, | 16.5) | (3.67, | 3.75) | (13.2, | 14.1) | (7.50, | 8.09) | (0.117, | 0.174) |
| **TITE-CRM** | **T2** | 14.0 | | 3.66 | | 18.3 | | 4.15 | | 15.4 | | 8.90 | | 0.118 | |
|  |  | (13.8, | 14.3) | (3.59, | 3.81) | (17.9, | 18.3) | (4.04, | 4.15) | (14.9, | 15.4) | (8.43, | 8.95) | (0.070, | 0.118) |
| **3+3** | **T2** | 11.1 | | 2.59 | | 23.2 | | 10.95 | | 19.7 | | 16.05 | | 0.127 | |
|  |  | (11.1, | 11.1) | (2.59, | 2.63) | (23.2, | 24.2) | (10.95, | 11.17) | (19.7, | 20.7) | (16.05, | 16.31) | (0.127, | 0.144) |
| **STARPAC** | **T3** | 13.2 | | 2.74 | | 17.1 | | 3.86 | | 13.9 | | 7.87 | | 0.468 | |
|  |  | (13.0, | 13.7) | (2.68, | 2.90) | (17.1, | 17.4) | (3.82, | 4.11) | (13.9, | 14.8) | (7.87, | 8.38) | (0.441, | 0.506) |
| **TITE-CRM** | **T3** | 13.0 | | 2.88 | | 17.0 | | 3.87 | | 14.0 | | 8.00 | | 0.637 | |
|  |  | (12.7, | 13.2) | (2.85, | 3.02) | (16.3, | 17.0) | (3.71, | 3.87) | (13.7, | 14.1) | (7.90, | 8.12) | (0.637, | 0.708) |
| **3+3** | **T3** | 11.9 | | 2.39 | | 25.3 | | 12.18 | | 21.6 | | 17.93 | | 0.165 | |
|  |  | (11.9, | 12.2) | (2.39, | 2.43) | (25.3, | 27.1) | (12.18, | 12.67) | (21.6, | 23.2) | (17.93, | 18.75) | (0.141, | 0.168) |
| **STARPAC** | **T4** | 15.0 | | 2.23 | | 19.5 | | 4.60 | | 15.8 | | 8.90 | | 0.728 | |
|  |  | (14.7, | 15.6) | (2.16, | 2.37) | (19.4, | 19.7) | (4.58, | 4.64) | (15.8, | 16.8) | (8.90, | 9.69) | (0.691, | 0.776) |
| **TITE-CRM** | **T4** | 11.4 | | 1.71 | | 14.6 | | 3.32 | | 12.3 | | 7.04 | | 0.895 | |
|  |  | (10.9, | 11.6) | (1.63, | 1.82) | (14.6, | 15.0) | (3.19, | 3.40) | (11.8, | 12.5) | (6.59, | 7.04) | (0.879, | 0.932) |
| **3+3** | **T4** | 13.2 | | 1.79 | | 29.2 | | 14.12 | | 24.9 | | 20.81 | | 0.456 | |
|  |  | (13.2, | 13.4) | (1.78, | 1.82) | (29.2, | 29.9) | (14.12, | 14.84) | (24.9, | 25.7) | (20.81, | 21.85) | (0.443, | 0.473) |
| **STARPAC** | **T5** | 12.8 | | 3.59 | | 16.5 | | 3.74 | | 13.7 | | 7.41 | | 0.444 | |
|  |  | (12.7, | 13.3) | (3.54, | 3.81) | (16.4, | 17.0) | (3.74, | 3.88) | (13.6, | 14.2) | (7.41, | 8.08) | (0.439, | 0.476) |
| **TITE-CRM** | **T5** | 16.6 | | 5.11 | | 21.6 | | 5.08 | | 18.2 | | 10.66 | | 0.347 | |
|  |  | (16.5, | 17.0) | (5.11, | 5.51) | (21.5, | 22.3) | (4.92, | 5.17) | (17.9, | 18.6) | (10.44, | 10.72) | (0.255, | 0.347) |
| **3+3** | **T5** | 9.9 | | 2.65 | | 20.4 | | 9.55 | | 17.3 | | 13.91 | | 0.349 | |
|  |  | (9.9, | 10.1) | (2.65, | 2.68) | (20.4, | 21.7) | (9.55, | 9.98) | (17.3, | 18.7) | (13.91, | 14.68) | (0.332, | 0.349) |
